# Supplementary material for: An overseas business paradox: Are Japanese general contractors risk takers?
Source: PLoS One. 2020 Sep 11;15(9):e0238570. doi: 10.1371/journal.pone.0238570 (PMC7485813; doi:10.1371/journal.pone.0238570)
Supplement: Attachment — Submitted filename: Ref1_1stR_PO_MKK_overseas_July_2_2020.pdf [file pone.0238570.s002.pdf]

Responses to referee I regarding the manuscript "An overseas business paradox: Are Japanese general contractors risk takers?" submitted to *PLoS ONE*

First, thank you for your comments and the time spent with our manuscript. Your constructive comments and suggestions improve our paper. We have tried to clarify all of your quarries and modified our paper according to your comments. Our responses to your comments are given below.

Referee I's general comments and our responses

This is an interesting analysis studying the overseas activity of Japanese general contractors. My comments are listed below:

Our response: Thank you for your comments and the time spent to evaluate our manuscript. We believe that your comments and suggestions really helped improve our manuscript. Following your comments, we have revised our manuscript. We have prepared the response to each of your comments in a way that you can clearly observe the modifications to the manuscript.

Referee I's specific comments and our responses

1. **Referee comment**: p. 16: is there any data on whether projects were completed fully or only partially completed? Or if projects took longer than expected. Those factors could ultimately influence the actual rate the contractor faces.

   Our response: The dataset of business operations is taken from the Overseas Construction Association of Japan (OCAJI). Although this dataset provides useful information about overseas projects, it contains the information of orders of overseas projects but not contain the information of whether projects were completed fully or only partially completed. We agree that disbursement-based information including fully/partially completion of projects is more reliable than our order-based information. Accordingly, we added footnote 18 in page 16 as follows:

   [***Page 10, footnote 18***: *Another possible problem is that our dataset contains the information of orders of overseas projects but not the information of whether projects were completed fully or only partially completed. These factors could ultimately influence the actual rate the contractor faces. Although we agree that disbursement-based information, including fully/partially completion of projects, is more reliable, we use the order-based information in our analysis due to the data limitation.*] ■

2. **Referee comment**: p. 17: as you note, there are a large number of zeros in your data. Have you considered using a zero-modified Poisson distribution in your analysis?

   Our response: Thank you for your helpful comments and suggestions. We totally agree with the reviewer's comment that zero-modified models should be applied to our model estimations. Accordingly, we conducted zero-inflated Poisson and negative binomial models and the Poisson pseudo-maximum likelihood (PPML) estimation. The results of these methods generally support our baseline results. We revised our manuscript by adding Tables 9-10 and revising the corresponding parts as follows.

[***Pages 10-13, lines 327-367***: *The OCC is a count variable capturing the number of orders of overseas contracts for each general contractor and each country. Many studies, including those on FDI location choice, have applied count data models (Smith Jr. and Florida, 1994, Wu, 1999, Coughlin and Segev, 2000, Zhou et al., 2002, Roberto, 2004, Yavan, 2010). This study applies Poisson models and negative binomial models (NBMs). The assumed equality of the conditional mean and variance can be considered the major shortcoming of the Poisson regression models. Among several alternatives, the most common is negative binomial models (NBMs). The NBM is an extension of the Poisson regression model by introducing an individual, unobserved effect into the conditional mean.*

One possible methodological problem is that since each general contractor has many countries where it has no operations, the dependent variable contains many zero counts, so that the distribution of the OCC data is skewed to the right and contains a large proportion of zeros (i.e., excess zeros). The data of no operations provides relevant information, since the independent variable containing zeros could help explain the reason why general contractors do not receive any orders of contracts in some specific countries. To deal with the problem related to the distributional characteristics, this study applies a zero-inflated Poisson and negative binomial regression models. The excess of zeroes is incorporated in zero-inflated models, which is a finite mixture model, where one distribution is considered as a degenerate process with a unit point mass at zero (Lambert, 1992). The model allows for excess zeros in count models under the assumption that the population is characterized by two groups: one group whose counts are generated by the Poisson or negative binomial model, and another group (absolute zero group) that have zero probability of a count greater than zero. Observed zero counts could come from either group. The likelihood of being in either group is estimated using a logit model (Long, 1997). The zero-inflated negative binomial model allows overdispersion through the splitting process that models the outcomes.

The OCA captures the total real value of the orders of overseas projects received by each contractor in each country. For this dependent variable, we apply ordinary least squares (OLS) for the estimation. However, similar to our count models, the model estimation may suffer from a zero problem, which is often discussed in the international trade literature, since OCA variable contains a significant portion of zero values. In our data set, for each contractor, there are many countries in which it does not receive any orders of contracts, as mentioned in the previous discussion. This kind of zero-contract amounts is considered as a corner solution outcome in the context of economic theory, where typical OLS estimation may not be appropriate. To mitigate this issue, we apply the Tobit model by using the log of one plus the total real value of the orders of overseas projects as the dependent variable. In addition, following the work of Santos Silva and Tenreyro (2006), we also apply the Poisson pseudo-maximum likelihood (PPML) estimations. The PPML estimation method can be applied to the levels of the total real value of the orders of overseas projects, rather than their log forms, to estimate directly the nonlinear form of the model. All estimated models include the year and contractor dummies to control for the year- and contractor-specific effects.] ■

We revised the first paragraph of subsection "Financial status in a credit market" as follows:

[***Page 17, lines 435-451***: *The result consistently shows that irrespective of the subsamples of low and high FIS, the coefficients on financial status (FIS) are significantly positive for all*

measures of overseas business operations, except for high FIS in the Poisson and negative binomial parts of the zero-inflated Poisson and negative binomial models and in the PPML model. It should be noted that the setup of the logit model in the zero-inflated models is to predict the probability of being no operations, so that a negative coefficient on an independent variable implies a positive relationship between the probability of operations and the independent variable. Since the high value of FIS implies the low evaluation in a credit market due mainly to the low profitability or the high default risk, the result suggests that less credible general contractors tend to expand overseas business operations by receiving orders of overseas projects. Given the argument that overseas business operations are risky in general, less credible general contractors tend to take a higher risk than highly credible ones. Moreover, tables 8 to 10 show that the estimated coefficients for the subsample of low FIS is larger than those for the subsample of high FIS. This implies that the measures of overseas business operations increase with a rise in FIS in a concave manner, i.e., the effect of financial status in credit markets on overseas business operations would decrease as financial status worsens.] ■

We revised the whole part of subsection "Other control variables."

[***Pages 22-24, lines 532-615****: Tables 8 to 10 also present the estimation results related to other control variables, CSIZE, ODA, FDI, ESIZE, CONC, INCM, and POLIT, all of which are expected to affect general contractors' location choice. Dividing full sample into the two subsamples of low FIS and high FIS allows us to verify the existence of the interaction effects of financial status and other control variables. The coefficients on the firm size (CSIZE), as another contractor-specific control variable, are significantly positive for all models in tables 8 to 10 and are significantly negative for the logit part of the zero-inflated models in table 9, which implies that large-sized general contractors tend to engage more in overseas business expansion. Possible justification for this result includes that large-sized general contractors implement projects in various fields of construction-related services so that they can comply with the requirement of projects' employers in foreign countries. In addition, the estimated coefficients on the firm size (CSIZE) for the subsample of low FIS is larger than those for the subsample of high FIS, which implies that the sensitivity of overseas business operations in response to the firm size is large for general contractors with high credibility in credit markets (low FIS).*

Concerning country-specific control variables, the coefficients on official development assistance (ODA) and foreign direct investment (FDI) in table 8 are significantly positive for most models, irrespective of the two subsamples of low FIS and high FIS. In addition, the coefficients on ODA and FDI in table 9 are positive and negative for the Poisson (negative binomial) part and the logit part of the zero-inflated models, respectively. Bilateral foreign aid by Japanese government and foreign investment by Japanese firms, particularly Japanese manufacturers, would encourage general contractors to expand overseas business operations. It is well known that one of the main targets of Japan's foreign aid is to promote infrastructure development in recipient countries. One possible obstacle for Japanese general contractors to receive the contract order is that under the current regulation of ODA from Japan, the tender procedure is open for any nationalities if the bidder satisfies the criteria given by executing agencies in the host country, even though the fund comes from Japanese govern-

Table 8: Locational choice of international operations

Variables	OCC						OCA					
	POISSON			NBREG			OLS			TOBIT		
	Full sample	Low FIS	High FIS	Full sample	Low FIS	High FIS	Full sample	Low FIS	High FIS	Full sample	Low FIS	High FIS
FIS	19.038***	105.353***	11.936	20.667***	91.679***	25.293***	1.790***	8.612***	1.120**	22.084***	102.565***	24.912***
	(4.986)	(21.811)	(9.395)	(6.857)	(23.073)	(10.220)	(0.530)	(3.021)	(0.572)	(8.380)	(32.086)	(11.994)
CSIZE	0.500**	1.483***	0.474***	1.010***	2.041***	1.061***	0.162***	0.205***	0.158***	1.876***	3.470***	2.001***
	(0.183)	(0.546)	(0.218)	(0.223)	(0.545)	(0.268)	(0.031)	(0.075)	(0.036)	(0.272)	(0.844)	(0.324)
ODA	0.092***	0.158***	0.015	0.108***	0.136***	0.085**	0.015***	0.018***	0.010*	0.164***	0.200***	0.107*
	(0.027)	(0.032)	(0.041)	(0.029)	(0.035)	(0.043)	(0.004)	(0.007)	(0.006)	(0.040)	(0.052)	(0.059)
FDI	0.254***	0.201***	0.336***	0.265***	0.188***	0.370***	0.052***	0.051***	0.053***	0.284***	0.163***	0.447***
	(0.030)	(0.033)	(0.051)	(0.023)	(0.025)	(0.039)	(0.004)	(0.007)	(0.005)	(0.035)	(0.043)	(0.057)
ESIZE	−0.042	−0.165***	0.067	−0.101**	−0.115**	−0.124*	−0.041***	−0.040***	−0.041***	−0.231***	−0.102	−0.384***
	(0.045)	(0.044)	(0.068)	(0.042)	(0.049)	(0.067)	(0.006)	(0.009)	(0.007)	(0.056)	(0.072)	(0.088)
INCM	−0.099	−0.270***	0.093	−0.225***	−0.290***	−0.131	−0.032***	−0.053***	−0.010	−0.338***	−0.446***	−0.174
	(0.071)	(0.082)	(0.115)	(0.069)	(0.085)	(0.106)	(0.008)	(0.013)	(0.009)	(0.101)	(0.134)	(0.149)
POLIT	0.001	0.002	−0.001	0.000	0.008	−0.007	0.002***	0.004***	0.001	0.013	0.028***	−0.006
	(0.005)	(0.006)	(0.009)	(0.006)	(0.007)	(0.010)	(0.001)	(0.001)	(0.001)	(0.009)	(0.011)	(0.013)
CONC	−3.695***	−4.490***	−2.888***	−3.802***	−4.358***	−3.360***	−0.649***	−0.864***	−0.438***	−6.338***	−6.832***	−5.622***
	(0.281)	(0.323)	(0.417)	(0.160)	(0.193)	(0.240)	(0.026)	(0.042)	(0.029)	(0.244)	(0.306)	(0.374)
Constant	−7.886***	−14.834***	−11.121***	−11.017***	−22.474***	−11.363***	−0.341	−0.939	−0.541	−19.617***	−40.704***	−17.319***
	(1.978)	(6.240)	(2.711)	(2.872)	(6.254)	(3.672)	(0.478)	(0.881)	(0.557)	(3.556)	(9.806)	(4.454)
Obs	16145	8046	8099	16145	8046	8099	16145	8046	8099	16145	8046	8099
R-squared	0.607	0.660	0.587	-	-	-	0.217	0.258	0.163	0.262	0.281	0.246

***, ** and * are significant at the $1\,\%$, $5\,\%$ and $10\,\%$ levels, respectively.
All models include contract and year dummies.
Robust standard errors are in parentheses.

Table 9: Locational choice of international operations: Zero-inflated Poisson and negative binomial regressions

Variables	Zero-inflated POISSON						Zero-inflated NBREG					
	Full sample		Low FIS		High FIS		Full sample		Low FIS		High FIS	
	POISSON	Zero inflated	POISSON	Zero inflated	POISSON	Zero inflated	NBREG	Zero inflated	NBREG	Zero inflated	NBREG	Zero inflated
FIS	8.591**	−19.835***	62.715***	−41.631***	1.754	−29.779***	15.358***	−14.571***	78.409***	−37.018**	10.597	−21.342***
	(4.168)	(3.847)	(23.483)	(13.331)	(7.271)	(5.162)	(5.910)	(4..882)	(24.936)	(16.314)	(8.808)	(6.658)
CSIZE	0.015	−0.964***	−0.066	−1.001***	−0.099	−0.754***	0.377	−0.938***	0.849	−0.970***	0.407	−0.658***
	(0.612)	(0.045)	(0.528)	(0.056)	(0.545)	(0.088)	(0.241)	(0.056)	(0.784)	(0.078)	(0.302)	(0.100)
ODA	0.036	−0.125***	0.064**	−0.160***	−0.013	−0.081**	0.047	−0.117***	0.065*	−0.149***	−0.011	−0.098*
	(0.025)	(0.025)	(0.029)	(0.035)	(0.039)	(0.036)	(0.031)	(0.033)	(0.039)	(0.046)	(0.053)	(0.051)
FDI	0.187***	−0.125***	0.194***	−0.059**	0.172***	−0.211***	0.183***	−0.111***	0.180***	−0.042	0.212***	−0.184***
	(0.028)	(0.023)	(0.033)	(0.029)	(0.041)	(0.037)	(0.026)	(0.029)	(0.033)	(0.039)	(0.042)	(0.045)
ESIZE	0.002	0.141***	−0.146***	0.045	0.161***	0.255***	−0.013	0.157***	−0.130	0.029	0.120	0.286***
	(0.041)	(0.036)	(0.050)	(0.049)	(0.057)	(0.054)	(0.062)	(0.055)	(0.088)	(0.089)	(0.076)	(0.069)
INCM	−0.096	0.195***	−0.119	0.227**	−0.005	0.136	−0.193***	0.127	−0.208***	0.150	0.065	0.199
	(0.070)	(0.067)	(0.082)	(0.092)	(0.117)	(0.101)	(0.092)	(0.097)	(0.106)	(0.127)	(0.171)	(0.155)
POLIT	−0.012**	−0.009*	−0.011*	−0.022***	−0.014*	0.005	−0.008	−0.009	−0.009	−0.024***	−0.002	0.010
	(0.005)	(0.005)	(0.006)	(0.007)	(0.008)	(0.008)	(0.006)	(0.007)	(0.007)	(0.009)	(0.010)	(0.011)
CONC	−0.959***	3.419***	−1.472***	3.721***	−0.550*	3.072***	−1.539***	3.266***	−2.127***	3.458***	−0.411	3.280***
	(0.245)	(0.173)	(0.353)	(0.249)	(0.309)	(0.259)	(0.250)	(0.234)	(0.344)	(0.337)	(0.331)	(0.319)
Constant	0.673***	10.488***	6.108	13.999***	−2.657	4.628***	−2.294	9.314***	−4.325	13.794***	−8.961***	1.358
	(1.915)	(1.190)	(6.095)	(1.621)	(2.657)	(1.922)	(2.294)	(1.571)	(8.644)	(2.400)	(4.084)	(2.456)
Obs	16145		8046		8099		16145		8046		8099	
Likelihood[1]	−8693.15		−4644.87		−3738.72		−6498.94		−3559.90		−2857.12	

***, ** and * are significant at the 1 %, 5 % and 10 % levels, respectively.
All models include contract and year dummies.
Robust standard errors are in parentheses.
[1] "Likelihood" denotes the log-pseudolikelihood for each estimation.

Table 10: Locational choice of international operations: Poisson pseudo-maximum likelihood (PPML) estimation where the dependent variable is the total real value of the orders for the overseas projects

Variables	Full sample	Low FIS	High FIS
FIS	31.710***	104.401***	11.743
	(9.907)	(41.755)	(11.407)
CSIZE	0.801***	1.353*	0.657**
	(0.230)	(0.801)	(0.300)
ODA	−0.004	0.045	−0.119**
	(0.042)	(0.058)	(0.048)
FDI	−0.020	−0.096***	−0.282***
	(0.034)	(0.034)	(0.062)
ESIZE	0.082	0.139**	−0.181**
	(0.050)	(0.059)	(0.082)
INCM	−0.468***	−0.629***	0.027
	(0.162)	(0.223)	(0.142)
POLIT	0.025***	0.022*	0.033***
	(0.010)	(0.013)	(0.013)
CONC	−4.310***	−4.520***	−3.368***
	(0.337)	(0.416)	(0.531)
Constant	−13.667***	−19.196	−5.563
	(3.116)	(11.743)	(4.239)
Obs	16145	8046	8099
R-squared	0.283	0.304	0.497

***, ** and * are significant at the 1 %, 5 % and 10 % levels, respectively.
All models include contract and year dummies.
Robust standard errors are in parentheses.

ment. Such a circumstance causes Japanese firms to face the intense competition against international bidders, especially Chinese and Korean firms with the cost-related advantage.[1] *However, some projects require advanced technology, and Japanese firms generally have the advantage in construction technology and experiences. Thus, some grant aid projects are the exceptions from the open tender system, so that only Japanese firms are eligible to implement these projects. The positive association of ODA with overseas business operations in our empirical analysis suggests the positive role of foreign aid from Japan in helping Japanese general contractors' expansion of their business to foreign countries, although the open tender system intensifies the competition with foreign contractors.*

In addition to foreign aid from Japan, the positive association of FDI with overseas business operations implies that direct investment of Japanese firms is also one of the crucial factors for Japanese general contractors' behavior. It should be noticed that the party to engage in foreign investment is not contractors themselves, but manufacturers, such as automobiles, electrical parts, textile, and retail dealers. Foreign investment of Japanese manufacturers creates business opportunities to Japanese general contractors. When Japanese manufacturers set up new factories or facilities, they often order new construction to Japanese general contractors although they are free to choose non-Japanese firms. This is due mainly to the motivation to mitigate various risk factors, including the construction period and the quality of buildings, through the long-term reliance established between general contractors and manufacturers. In particular, the manufacturers that start business in a specific country without proper knowledge and information tend to order Japanese general contractors as a kind of inward security.

The comparison of the estimated coefficients on ODA and FDI for the two subsamples generally suggests that the coefficients on ODA for the subsample of low FIS are larger than those for the subsample of high FIS, while the coefficients on FDI for the subsample of low FIS are smaller than those for the subsample of high FIS. When ODA from Japan increases, general contractors with high credibility (low FIS) tend to expand their overseas business operations more aggressively, compared to general contractors with low credibility (high FIS). In contrast, when FDI from Japan increases, general contractors with low credibility (high FIS) tend to expand their overseas business operations more aggressively, compared to general contractors with high credibility (low FIS). These results illustrate that credible general contractors with advantageous financing costs tend to receive the orders of relatively less risky ODA-related projects, while less credible general contractors tend to receive the orders of projects financed through direct investment by Japanese firms.

For other country-specific control variables, the analysis presents that the size of economy (ESIZE) are negatively associated with overseas business operations, although some models show less clear or inconsistent results. This result partly supports that Japanese general contractors tend to expand their overseas business operations in small-sized countries. In addition, per capita income (INCM) is negatively associated with overseas business opera-

[1] Another problem is the financing issue related to the fact that for most of infrastructure development, covering all costs through ODA is almost impossible. Thus, Japanese firms are recommended to establish new business schemes, including operation after completion of the construction, and other alternative financing schemes, such as Public Private Partnership (PPP), where private business venture is often funded and operated through a partnership of the recipient government and private enterprises.

tions for the full sample and the subsample of low FIS, while there is no clear relationship between them for the subsample of high FIS. This implies that general contractors, particularly credible general contractors, are reluctant to expand their overseas business operations to low-income countries. Moreover, political stability (POLIT) is positively associated with overseas business operations particularly for the subsample of low FIS, although some models show insignificant results. This result suggests that credible general contractors are likely to pay more attention to political stability of the country when they expand their overseas business operations. Finally, the concentration measure (CONC) is negatively correlated with overseas business operations, so that Japanese general contractors are likely to expand their overseas business to the countries where other general contractors have already been under operations. In other words, Japanese general contractors may be characterized as a follower of other successful firms in each country. The larger value of the estimated coefficients on CONC for the subsample of low FIS implies that this tendency would be more substantial for credible general contractors.] ■

3. **Referee comment**: What level of competition exists in bids? Why are some overseas projects willing to accept a bid from a higher risk contractor. Can you explain this process more.

   Our response: Thank you for your comments. As we explained in subsection [3.3.1 Financial status in a credit market], general contractors with weak financial conditions tend to expand their overseas business than those with strong financial conditions. Administrators of overseas projects usually prefer to contract with financially strong general contractors in the bidding process. However, since overseas projects, mostly in developing countries, are perceived as high-risk projects and less profitable than domestic projects, financially strong general contractors seem to be reluctant to participate in competitive bidding or to set a very high price in competitive bidding. On the other hand, financially weak general contractors seem to be eager to take such high risk partly to keep their operations and employment. We revised our manuscript by adding this point as follows:

   [***Page 21, lines 492-499***: *Administrators of overseas projects usually prefer to contract with financially strong general contractors in the bidding process. However, since overseas projects, mostly in developing countries, are perceived as high-risk projects and less profitable than domestic projects, financially strong general contractors seem to be reluctant to participate in competitive bidding or to set a very high price in competitive bidding. On the other hand, financially weak general contractors seem to be eager to take such high risk partly to keep their operations and employment.*] ■

We finally revised the whole part of subsection 3.3.2 [Other control variables] as follows:

4. **Referee comment**: Do you have data for these contractors on their domestic projects so that comparisons can be made on their actual interest rates for domestic versus overseas projects?

   Our response: Thank you for your comments. We think that the comparison between domestic and overseas projects is needed to verify our results. However, due to the unavailability of the detailed data of domestic and overseas projects, such analyses are difficult in our empirical framework. We revised our manuscript by adding this point as a footnote:

[***Page 21, footnote 20***: *To verify this argument related to domestic and overseas projects, we need more careful analyses, including the comparison between domestic and overseas projects. However, due to the unavailability of the detailed data of domestic and overseas projects, such analyses are difficult in our empirical framework. The careful examination will be left for future research.*] ■

References

Coughlin, C. and Segev, E. (2000). Foreign direct investment in China: A spatial econometric study. *World economy*, 23:1–23.

Lambert, D. (1992). Zero-inflated Poisson regression, with an application to defects in manufacturing. *Technometrics*, 34:1–14.

Long, J. (1997). *Regression models for categorical and limited dependent variables*. Sage.

Roberto, B. (2004). Acquisition versus greenfield investment: The location of foreign manufacturers in Italy. *Regional science and urban economics*, 34:3–25.

Santos Silva, J. and Tenreyro, S. (2006). The log of gravity. *Review of economics and statistics*, 88:641–658.

Smith Jr., D. and Florida, R. (1994). Agglomeration and industrial location: An econometric analysis of Japanese-affiliated manufacturing establishments in automotive-related industries. *Journal of urban economics*, 36:23–41.

Wu, F. (1999). Intrametropolitan FDI firm location in Guangzhou, China: A poisson and negative binomial analysis. *Annals of regional science*, 33:535–555.

Yavan, N. (2010). The location choice of foreign direct investment iithin Turkey: An empirical analysis. *European planning studies*, 18:1675–1705.

Zhou, C., Delios, A., and Yang, J. (2002). Locational determinants of Japanese foreign direct investment in China. *Asia Pacific journal of management*, 19:63–86.
